# Cadmium adsorption using novel MnFe_2_O_4_-TiO_2_-UIO-66 magnetic nanoparticles and condition optimization using a response surface methodology

**DOI:** 10.1039/c9ra03430g

**Published:** 2019-06-27

**Authors:** Pedram Nasehi, Boshra Mahmoudi, Seyed Foad Abbaspour, Mojtaba Saei Moghaddam

**Affiliations:** Department of Chemical Engineering, Quchan University of Technology Quchan Iran mojtabasaei@qiet.ac.ir mojtabasaei@gmail.com; Research Centre, Sulaimani Polytechnic University Sulaimani 46001 Kurdistan Region Iraq

## Abstract

In this study, magnetic nanocomposites (UIO-66-MnFe_2_O_4_-TiO_2_) were synthesized based on the metal–organic framework. To investigate the synthesized adsorbent structure, XRD, SEM, FT-IR, BET, and VSM techniques were used, and also an EDX test was applied after adsorption of Cd(ii). The synthesized nanocomposite was used for Cd(ii) adsorption. The effects of four parameters such as the amount of adsorbent (0.05 to 0.25 g), pH (1 to 9), adsorption time (24 to 120 minutes), initial amount of metal ion (100 to 900 mg), at five levels (−2 to +2) were evaluated during the experiment based on the Response Surface Methodology (RSM) using Central Composite Design (CCD) and then, the optimal levels were determined. The *F*-value and *P*-value of the fitted second order model were obtained as 6039.62 and 0.0001, respectively. Optimization of the values was also performed for variables and, initial concentration of metal, pH, adsorption time and amount of adsorbent were obtained as 5.2 mg l^−1^, 5, 63 minutes, and 0.18 g, respectively as optimum conditions. For optimal conditions, the maximum adsorbance was equal to 98%. Investigation of kinetic and isotherm adsorption showed that a second-order kinetic model and a Langmuir isotherm cover the Cd(ii) adsorption data well. It was also revealed that the adsorbent was removed from the environment by an external magnetic field.

## Abstract 2

In this study, magnetic nanocomposites (UIO-66-MnFe_2_O_4_-TiO_2_) were synthesized based on the metal–organic framework.

## Graphical abstract

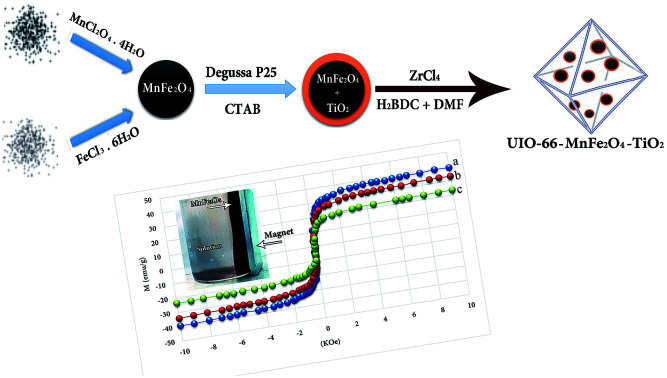

## 1 Introduction

The pollution caused by heavy metals is one of the main environmental problems in today's industrial world.^1^ Industrial waste is the most important source of heavy metals in the world. This waste has caused countless problems for the environment, especially aquaculture.^2^ According to reports of the World Health Organization (WHO), among the world's heaviest metals, cadmium in the form of Cd(ii) is one of the most dangerous, amounts of which should be very low. According to official statistics, the limit for cadmium in drinking water is set to a maximum 0.005 mg l^−1^.^3^ Main sources and special applications of cadmium, include Ni–Cd batteries, phosphate fertilizers, pesticides, paints, oil and gas refineries, welding and soldering, photography, plastics, iron and steel and cement manufacturing industries.^3,4^ Cadmium is one of the few elements having no constructive role in the human body, and its low concentrations in the human body cause irreparable problems.^5^ Considering the many problems for the environment caused by heavy metals, they should be removed from the environment by reliable means. Various methods have been proposed for removal of heavy metals from various sources among which, one can mention methods such as chemical processes, physical processes, oxidation processes, filtration and adsorption.^6^ Among the various methods used to remove heavy metals from different sources, adsorption is one of the most promising due to its specific properties such as high efficiency, low cost, and ease.^7^

Today, there are some of the world's best surfactants, and Metal–Organic Frameworks (MOFs) have a special position due to their special parameters. This materials because of their high porosity have acquired interesting features such as high adsorption capacity, synthesis ability, high applicability and high thermal resistance.^8^ Uio-66 is a MOF with chemical formula of Zr_6_O_4_(OH)_4_. MOFs with Zr core were first created by Kowka *et al.* and introduced to the world.^9^ Many features mentioned in favor of the MOFs specially UiO-66 along with its high thermal stability has caused these organic–metal frames become a special tool for adsorption; but to date, they have been widely used for adsorption of gases and in some sources, non-gas adsorption is noted.

Unlike other common sorbents such as zeolites, active carbon *etc.*, the chemical and structural properties of MOFs can completely change due to the selection of various components changing the metal clusters and organic ligands of these structures, which is a very interesting feature of organic–metal frames in adsorption is that.^10^ The ability of adsorbent modification is an important factor in selection of adsorbent. Among the various materials, MOFs have a particular priority due to their modifiability. The possible modifications to superficial interactions, and electrostatic interactions are very important. Because, electrostatic interactions are predictable through zeta potential and play a significant role in surface adsorption. Another remarkable feature of the MOFs is their high porosity and very high surface area which is one of the main pillars regarding selection of adsorbents.^11^ Main important features of a good adsorbent are ease of use and its attractive ability after adsorption. Today, magnetization is one of the best available techniques for separation of adsorbents after adsorption procedure. In that sense materials are added to them to find magnetic properties. MnFe_2_O_4_, a nanoscale material is one of the available materials used for magnetizing the adsorbents. These nanomaterials also cause magnetic properties in UIO-66 in addition to having adsorption properties. In addition to its adsorption ability, MnFe_2_O_4_ produces a much better magnetic surface than other nanomaterials such as MgFe_2_O_4_, CoFe_2_O_4_, ZnFe_2_O_4_, and NiFe_2_O_4._ This feature, coupled with the low cost, non-toxicity and applicability of this nanoparticle has led to a special application in adsorption.^12,13^ Coupling of this nanoparticle with TiO_2_ has been reported in the various sources to facilitate the adsorption and transport of nanoparticles. Besides simplifying the process, it increases the amount of adsorption due to the presence of free electrons in the TiO_2_ compound. In this paper, titanium oxide is shown to be a potentially excellent carrier for MnFe_2_O_4_ nanoparticle, and finally, a very simple and practical way of coupling TiO_2_-MnFe_2_O_4_-UIO-66 nanocomposite is proposed with a very high ability to separate cadmium from wastewater, and it can well be separated from the solution after adsorption by the magnetic field. In this study, adsorption of Cd(ii) ions from adsorbent was done by the help of the properties such as specific surface area, porosity, adsorption capacity and high adsorption rate, as well as modifying ability. MOFs are among the few materials having all of these features. But the basic obstacle regarding the use of many MOFs is their instability in aqueous solutions since their performance greatly reduces in aqueous solutions. Some of MOFs that are stable in aqueous solutions are highly hydrophobic. For example, ZIF-8 has no significant uptake of water near saturation vapour pressure. Among these materials, UIO-66 is water-friendly, besides it is stable in aqueous solutions and has prominent properties such as surface area, porosity, adsorption capacity and high adsorption rate, and easy to modify.^14^ Therefore, this substance was used for adsorption in this study. The adsorbent separation after the adsorption process is one of the important issues in the adsorption process. One of the great methods for separation is using a magnetic field, in which the adsorbent must have a magnetic property. MnFe_2_O_4_ is one of the high-adsorption magnetic materials, and was selected for placement in the UIO-66 structure due to the features such as ease of preparation, high resistance against external agents and environmental conditions, high magnetism, low cost, and high flexibility. But the major problem with magnetic materials during loading and moving them is their tendency to sintering, making it difficult to enter the structure of UIO-66. To solve this problem, TiO_2_ was used because of its good ability to form core–shell with MnFe_2_O_4_.^13,15^

In addition to its high chemical and thermal stability, TiO_2_ improves loading efficiency and ease of use on the adsorbent. This surface modification for MnFe_2_O_4_ made it easy to enter the UIO-66 structure, making it a great adsorbent.

## 2 Experiments

### 2.1 Materials and methods

In this research, materials were prepared from the precursor materials prepared by the German companies Merck and Evonik. During the experiment, the ionizing water made by the Kimia Tehran Acid Company was used. All materials in the tests were of high quality and were used without refinement.

### 2.2 Adsorbent UIO-66-MnFe_2_O_4_-TiO_2_ synthesis

#### 2.2.1 MnFe_2_O_4_ synthesis

A simple co-precipitation method was used to synthesize MnFe_2_O_4_. At first, 10.812 (gr) of (FeCl_3_·6H_2_O) With 3.958 (gr) of (Mn(Cl)_2_·4H_2_O) with a molar ratio (Fe : Mn = 2 : 1), were mixed 1 in 300 ml water ionized at 80 °C according to the below chemical eqn (1), then to adjust pH and reach to pH 12, 2 M NaOH solution was used. The solution was transferred to an oven with nitrogen atmosphere and was stirred at 80 °C by a magnetic stirrer. After changing the color of the solution from orange to black, it was removed from the oven and the MnFe_2_O_4_ sediment formed in black was removed by an external magnet. For complete purification of the precipitate, it was washed several times with deionized water and ethanol. Then the MnFe_2_O_4_ nanoparticles were dried in an oven at 70 °C for 12 hours and then heated to 500 °C for 4 hours in furnace.

1 MnCl_2_ + 2FeCl_3_ + 8NaOH → MnFe_2_O_4_ + 8NaCl + 4H_2_O

#### 2.2.2 Synthesis of MnFe_2_O_4_-TiO_2_

The synthesis of MnFe_2_O_4_-TiO_2_ was done by ultrasonic method and UW room. For this purpose, the ultrasonic pool was used. For TiO_2_ doping on MnFe_2_O_4_, the Degussa P25 precursor prepared was applied. Initially, 0.15 grams of synthesized MnFe_2_O_4_, with 0.15 grams of cetyltrimethyl ammonium bromide (CTAB), to 135 ml of *N*-butanol and pure ethanol solution (8 to 1). The solution was centrifuged at a speed of 6000 rpm for 30 minutes. After centrifugation, a few drops of 2% nitric acid solution were added. Then add 0.76 g Degussa P25 solution and transfer the solution into ultrasonic bath under ultraviolet radiation, adjust the bath temperature to 75 °C, and put it in the same conditions for 4 hours. The solution is then separated by centrifugation and the precipitate formed is washed several times with deionized water and ethanol. The precipitate was dried at 100 °C for 24 hours. Finally, it is heated to 500 °C for 2 hours. The optimal values for MnFe_2_O_4_-TiO_2_ synthesis were derived from the following reference and corrected in the manuscript.^15^

#### 2.2.3 Synthesis of UIO-66-MnFe_2_O_4_-TiO_2_

**Fig. 1:**
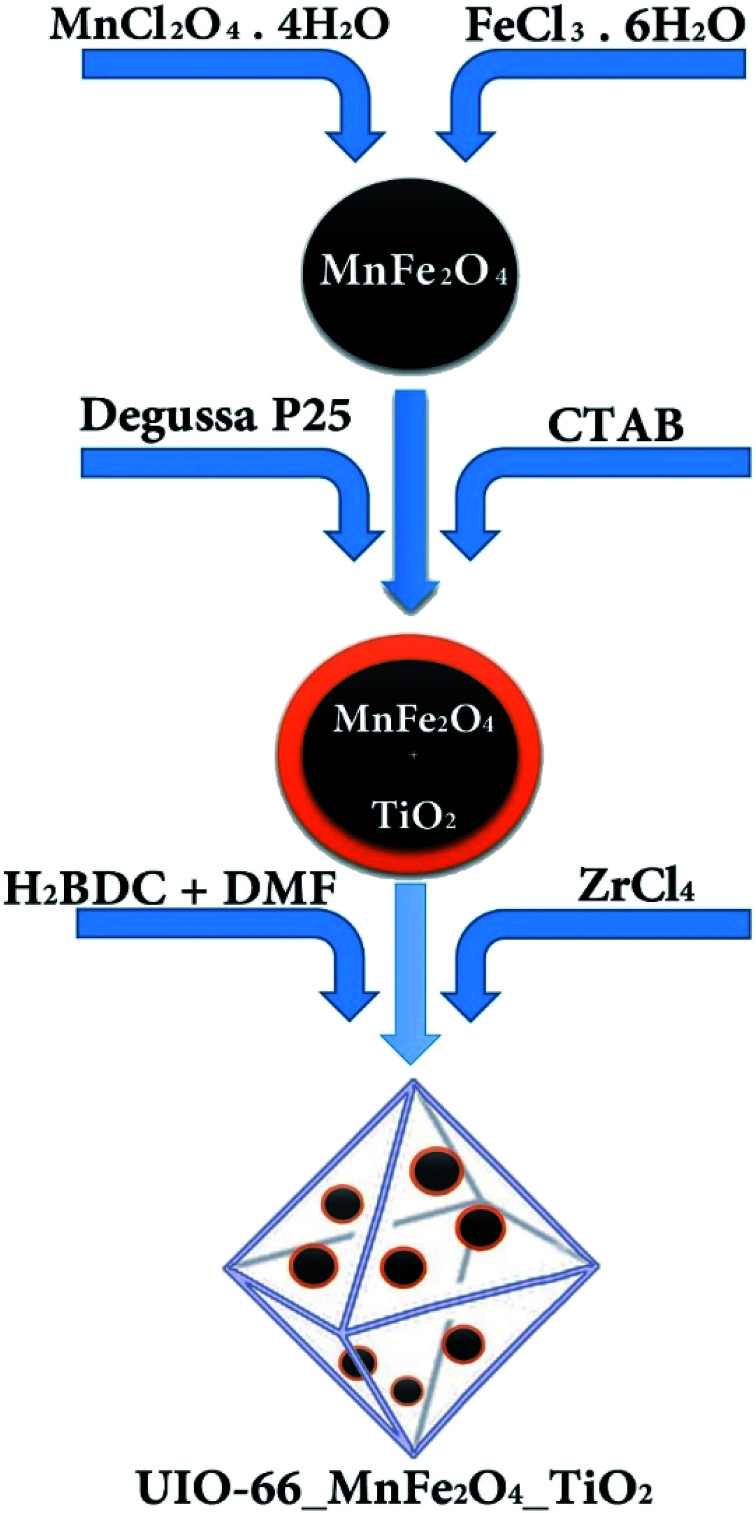
Schematic of adsorbent synthesis method.

To synthesize UIO-66-MnFe_2_O_4_-TiO_2_, the pre-synthesized substance of MnFe_2_O_4_-TiO_2_ was added to the compound synthesis of UIO-66. Hydrothermal method was used to synthesize UIO-66. In this method, initially, 0.424 gr of (ZrCl_4_) and 0.272 gr of 4 and 1 benzene dicarboxylic (H_2_BDC) with 0.135 gr of the synthesized MnFe_2_O_4_-TiO_2_ were dissolved in 200 gr of dimethylformamide (DMF) on a magnetic stirrer, then was transferred into an autoclave,and was placed in an oven at 120 °C for 24 hours. For activation of the produced material, the formed crystals were washed well with dimethyl solvent and then, were immersed in chloroform solution for 4 days, and dimethyl was replaced with the chlorometric solvent. To dry the crystals and remove the final solvent from the structure of the organic–metal frame, initially it was placed within it for 24 hours at a temperature of 190 °C. In order to synthesize the final composite according to the experimental design used in the paper and also to reduce the number of variables, some of the variables questioned by screening were reduced. One of these variables was the ratio of material variation that after synthesizing composite samples with different composition of MnFe_2_O_4_/TiO_2_ it was found that in low composition of loading, the magnetic power severely reduced. On the other hand, with the increase of MnFe_2_O_4_-TiO_2_ in the composite, the risk of forming UIO-66 goes up, so the best ratio for making composites in the manuscript has been reported (Fig. 1).

#### 2.2.4 Fabrication of contaminated metal samples

To create a polluting sample cadmium nitrate with a chemical formula Cd(NO_3_)_2_·4H_2_O was used. For the production of samples with different concentrations, an initial sample was used which was diluted for different tests.

### 2.3 Characterization of adsorbent

#### 2.3.1 X-Ray spectrometry (XRD)

**Fig. 2:**
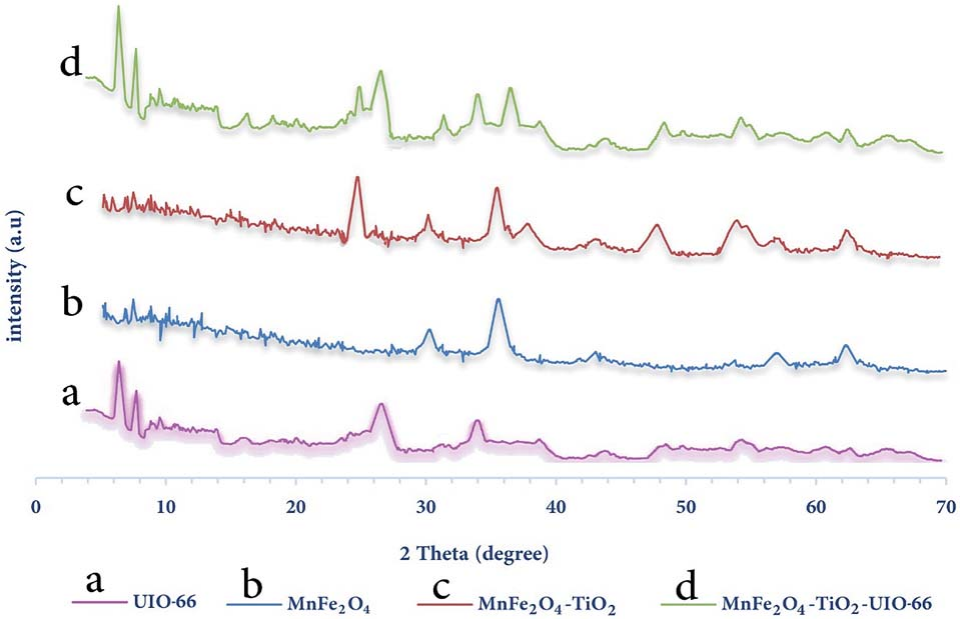
XRD pattern for synthesized samples (a) MnFe_2_O_4_, (b) MnFe_2_O_4_-TiO_2_, (c) UIO-66-MnFe_2_O_4_-TiO_2_.

In order to investigate the synthesized adsorbent structure, X-ray diffraction (XRD) performed and its results are shown in Fig. 2.

In Fig. 2, the XRD analysis was performed with the Philips PW 1840 XRD-manufactured by Phillips Netherlands. The Kα reflection from Cu (*λ* = 1.78897 Å) is in the 40 KV and 30 mA conditions. XRD images are registered from monitor mode (2*θ*) 5 to 70 degrees at angular velocity 0.02 (° sec^−1^). For example, MnFe_2_O_4_ in Fig. 2(a) peaks at 2*θ* = 30, 35, 43, 57 and 62 indicate the formation of this material. In Fig. 2(b), peaks of 2*θ* = 25, 38, 48 and 54 also show the presence of TiO_2_ in the MnFe_2_O_4_-TiO_2_ composition. Finally, in Fig. 2(c), the peaks of 2*θ* = 7, 9 and 27 indicate UIO-66 in the composition of MnFe_2_O_4_-TiO_2_-UIO-66 and the other peaks in Fig. 2 are about to the rest of the material mentioned above.

#### 2.3.2 FTIR spectrum

**Fig. 3:**
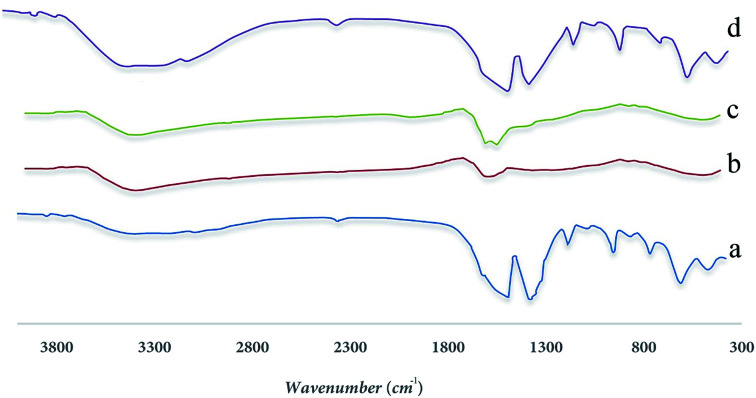
FTIR spectra for synthesized samples (a) UIO-66, (b) MnFe_2_O_4_, (c) MnFe_2_O_4_-TiO_2_, (d) UIO-66-MnFe_2_O_4_-TiO_2_.

An IR converter with an instant conversion made by BRUKER and ALPHA model was used to investigate functional groups in adsorbent using FTIR analysis in the range of 300 cm^−1^ to 3800 cm^−1^. Because each group has a unique band, it can be used to identify the functional groups and its associated peak, the results are shown in the Fig. 3 completely.

In Fig. 3(a), according to the results obtained from the FTIR spectrum taken from the Uio-66 allophilic framework, there is a peak in the region of 1521.05 cm^−1^ and another peak at 1407.35 cm^−1^, indicating two groups of carboxylates belonging to 4 and 1 benzene dicarboxylate attached to the zirconium metal from its O side. A peak has been reported in the region of 1193.32 cm^−1^ that indicates the benzene ring present in the composition of the BDC structure contained in the 66-Uio composition. Two peaks in the region of 1521.05 cm^−1^ and 1407.35 cm ^−1^ correspond to two carbon–oxygen bonds, the first one has a carbon dioxide double bond, located in the peak of 1525.37 and the other has a single carbon–oxygen bond, which is in 1407.35 cm^−1^ zone.

Fig. 3(b) corresponds to the MnFe_2_O_4_ material, and peaks from 500 to 600 cm^−1^ correspond to metal Fe–O, Mn–O bands. In Fig. 3(c), peaks of 1426 cm^−1^, 1641 cm^−1^, 2926 cm^−1^ indicate the bands contained in the TiO_2_ substance, and finally, in Fig. 3(d), the peaks observed indicate the total of the above bands in the MnFe_2_O_4_-TiO_2_-UIO-66.

#### 2.3.3 Investigate the magnetic properties

**Fig. 4:**
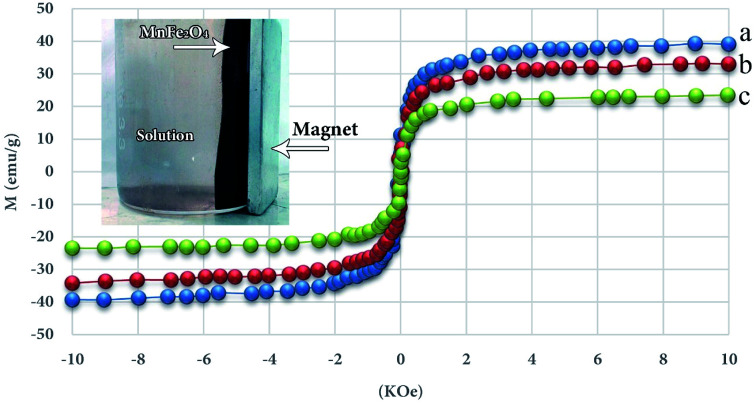
Magnetic curves for MnFe_2_O_4_ (a), MnFe_2_O_4_-TiO_2_ (b) and-MnFe_2_O_4_-TiO_2_ UIO-66 (c).

As shown in Fig. 4, all specimens exhibit an extremely paramagnetic characteristic, and pure MnFe_2_O_4_, MnFe_2_O_4_-TiO_2_ and UIO-66-MnFe_2_O_4_-TiO_2_ have a magnetic saturation value of 39.2, 33.4 and 23.5 emu g^−1^, the addition of TiO_2_ alongside MnFe_2_O_4_, MnFe_2_O_4_-TiO_2_ has a lower magnetic saturation than MnFe_2_O_4_, and when the UIO-66 couples, magnetic power decreases in advance, but it should be noted that the UO-66-MnFe_2_O_4_-TiO_2_ nanocomposite still has strong magnetic strength, which indicates its ability to separate magnetic and recovery.

#### 2.3.4 Surface area (BET analysis)

To determine the surface area of the synthesized samples, the Surface Area & Porosity Analyzer (BET) of the Micromeritics Company, TriStar II PLUS, was used and the porosity was measured using a nitrogen adsorbent isotherm at 77 °C. The measured values was shown in the Table 1.

**Table 1:** The surface area of the synthesized samples

Sample	*S*_BET_ [m^2^ g^−1^]
MnFe_2_O_4_	94.1
MnFe_2_O_4_-TiO_2_	112.3
MnFe_2_O_4_-TiO_2_-UIO-66	792
UIO-66	1167.16

#### 2.3.5 Scanning electron microscope (SEM) of the adsorbent

**Fig. 5:**
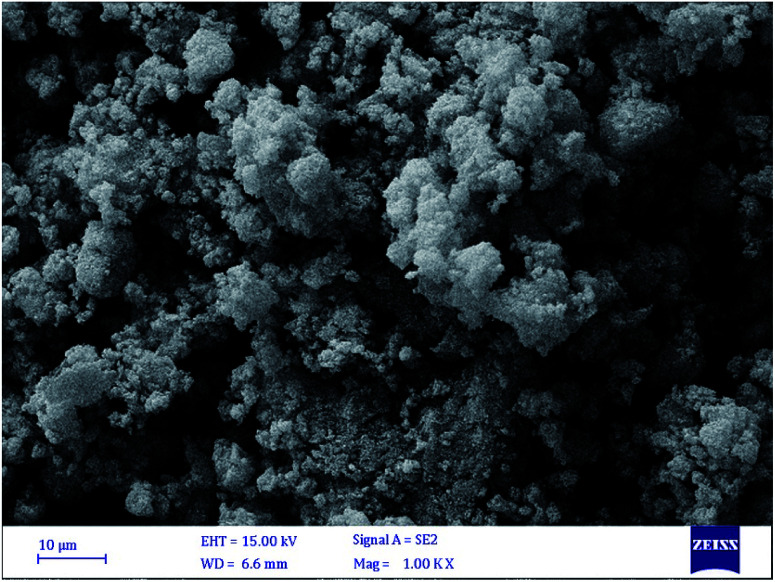
The SEM image of synthesized sample.

In order to investigate the adsorbent surface of the synthesized adsorbent sample, the SEM image was taken and it was shown in the Fig. 5.

### 2.4 Experiment design

In this research, the design of the experiment was used to carry out experiments by using Design Expert Software Version 11. Four important operational parameters including pH, adsorbent amount initial metal ion concentrations and test time were investigated in 5 different levels. The operational parameters for the experiment design are shown in Table 2 (in the table *a* – the initial metal ion concentration, *b* – pH, *c* is the adsorption time and *d* is the adsorbent amount). In the Table 2, the variables (*a*–*d*) and the levels of each were expressed in the 5 levels.

**Table 2:** Coded levels and values of experimental design

Levels	Variables			
	*a* – initial metal concentration (mg L^−1^)	*b* – pH	*c* – absorption time (min)	*d* – absorbent amount (gr)
2	900	9	120	0.25
1	700	7	96	0.2
0	500	5	72	0.15
−1	300	3	48	0.1
−2	100	1	24	0.05

Eqn (2) was used to study the relationship between the values obtained from experiments and modeling.2
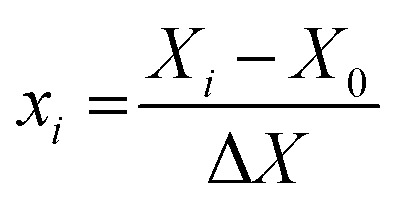
where, *x*_*i*_ is the dimensionless coded value of the *i*^th^ independent variable, *X*_*i*_ is the measured value of the independent variable, *X*_0_ is the value of *X*_*i*_ at the center point and the step change value is Δ*X*. All runs were performed in triplicate. Using RSM, a quadratic polynomial equation (eqn (3)) was considered for the prediction of adsorption percentage (response) as a function of independent variables (*a*–*d*) and their interactions.3
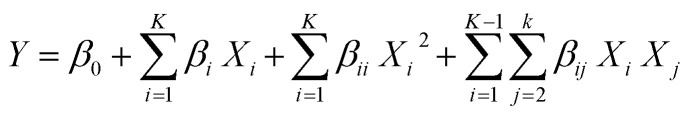
where, *Y* is the predicted value, *β*_0_, *β*_*i*_, *β*_*ii*_ and *β*_*ij*_ are constant regression coefficients of quadratic model, *X*_*i*_ and *X*_*j*_ represented the independent variables. In order to examine the variables, 31 tests were used as shown in Table 3. The equations were validated by ANOVA (analysis of variance). The two-dimensional and three-dimensional views of the software were helpful to check the independent variables effects. To determine the adsorption rate of Cd(ii), the UV/Vis spectrophotometer manufactured by UNICO UV/VIS 4800 was used. Eqn (4) was applied to calculate the adsorption percentage of Cd(ii).4
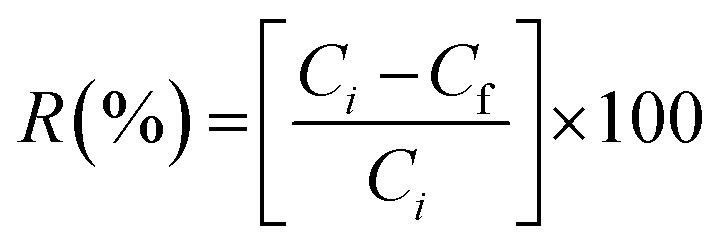
where, *C*_*i*_ is the initial concentration of metal (mg l^−1^) and *C*_f_ is the metal concentration after adsorption time (min).

**Table 3:** CCD matrix showing coded and real values of factors and adsorption of Cd(ii)

Row	The experiment priorities	Software parameters (*a*–*d*)				Actual values used in the experiment				Result obtained by adsorbent
		*a*	*b*	*c*	*d*	*a* – initial metal concentration (mg l^−1^)	*b* – pH	*c* – absorption time (min)	*d* – adsorbent amount (mg)	Absorption percentage (%)
1	4	1	1	−1	−1	700	7	48	0.1	40.81
2	21	0	0	−2	0	500	5	24	0.15	87.76
3	29	0	0	0	0	500	5	72	0.15	98.31
4	12	1	1	−1	1	700	7	48	0.2	38.81
5	31	0	0	0	0	500	5	72	0.15	98.39
6	6	1	−1	1	−1	700	3	96	0.1	75.51
7	23	0	0	0	−2	500	5	72	0.05	90.21
8	2	1	−1	−1	−1	700	3	48	0.1	90.66
9	28	0	0	0	0	500	5	72	0.15	98
10	22	0	0	2	0	500	5	120	0.15	94.51
11	8	1	1	1	−1	700	7	96	0.1	33.85
12	15	−1	1	1	1	300	7	96	0.2	49.86
13	10	1	−1	−1	1	700	3	48	0.2	65.81
14	20	0	2	0	0	500	9	72	0.15	95.06
15	14	1	−1	1	1	700	3	96	0.2	42.46
16	11	1	−1	1	−1	700	3	96	0.1	38.01
17	13	−1	−1	1	1	300	3	96	0.2	81.31
18	26	0	0	0	0	500	5	72	0.15	98.32
19	25	0	0	0	0	500	5	72	0.15	98.05
20	30	0	0	0	0	500	5	72	0.15	98.68
21	17	−2	0	0	0	100	5	72	0.15	69.45
22	3	−1	1	−1	−1	300	7	48	0.1	78.46
23	16	1	1	1	1	700	7	96	0.2	39.11
24	27	0	0	0	0	500	5	72	0.15	97.98
25	9	−1	−1	−1	1	300	3	48	0.2	78.51
26	18	2	0	0	0	900	5	72	0.15	86.91
27	7	−1	1	1	−1	300	7	96	0.1	68.86
28	1	−1	−1	−1	−1	300	3	48	0.1	86.96
29	5	−1	−1	1	−1	300	3	96	0.1	79.56
30	24	0	0	0	2	500	5	72	0.25	90.01
31	19	0	−2	0	0	500	1	72	0.15	86.81

## 3 Result and discussion

### 3.1 Analysis of the experiment design

Experimental data were used to calculate the predicted response by the software. The quadratic polynomial eqn (5) was obtained between the responses obtained in experiments and independent variables.

5 *Y* = 98.52 + 4.36*a* − 0.1875*b* − 0.3125*c* − 3*d* − 3.37*ab* − 0.3781*ac* − 0.7031*ad* + 5.091*bc* − 0.5219*bd* + 4.62*cd* − 5.09*a*^2^ − 0.7716*b*^2^ − 0.8466*c*^2^ − 0.2279*d*^2^ − 0.7656*abc* + 8.57*abd* − 4.3*acd* − 1.0*bcd* − 12.08*a*^2^*b* − 5.36*a*^2^*c* − 6.17*a*^2^*d* − 11.35*ab*^2^ + 2.59*abcd* − 30.88*a*^2^*b*^2^ + *ε*

where, *Y* is the percentage of pollutant removal, *a*, *b*, *c*, and *d* are independent variables, respectively (initial concentration of metal, pH, test time, adsorbent amount) and *ε* is also the standard error rate. The variance level of ANOVA is calculated and shown in Table 4.

**Table 4:** Analysis of variance (ANOVA) for response surface model for of adsorption of Cd(ii)

Row	Parameter	Sum squares	df	*F* value	*P* value
1	Model	16 766.70	24	6039.62	0.0001
2	A-A	152.43	1	1317.04	0.0017
3	B-B	0.2813	1	2.29	0.184
4	C-C	0.7813	1	6.29	0.0484
5	D-D	0.7200	1	6.42	0.0401
6	AB	181.75	1	1572.33	0.0001
7	AC	2.51	1	19.30	0.0041
8	AD	7.66	1	68.37	0.0001
9	BC	414.43	1	3584.84	0.0001
10	BD	4.38	1	37.24	0.0009
11	CD	341.68	1	2954.66	0.0001
12	A^2^	643.23	1	5563.58	0.0001
13	B^2^	14.59	1	128.67	0.0001
14	C^2^	17.23	1	154.26	0.0001
15	D^2^	1.53	1	11.17	0.015
16	ABC	9.93	1	81.08	0.0001
17	ABD	1157.63	1	10 162.72	0.0001
18	ACD	300.59	1	2594.43	0.0001
19	BCD	16.10	1	139.15	0.0001
20	A^2^B	778.03	1	6725.68	0.0001
21	A^2^C	153.10	1	1324.24	0.0001
22	A^2^D	203.16	1	1756.19	0.0001
23	AB^2^	686.58	1	5938.55	0.0001
24	ABCD	107.38	1	928.25	0.0001
25	A^2^B^2^	5086.75	1	5086.25	0.0001

In Table 4, the coefficients *F* and *P* values are calculated by the software are shown. Large *F* values and small *P* values indicate the significance of the respective coefficients. The most reliable way for the evaluation of the fitted model is using ANOVA, which can be used to compare the quality of the responses between obtained in the model and the experimental data responses. The effect of the parameters *a*–*d* on variance and adsorption chart determines that all four parameters examined have a significant effect on the adsorption procedure.

**Fig. 6:**
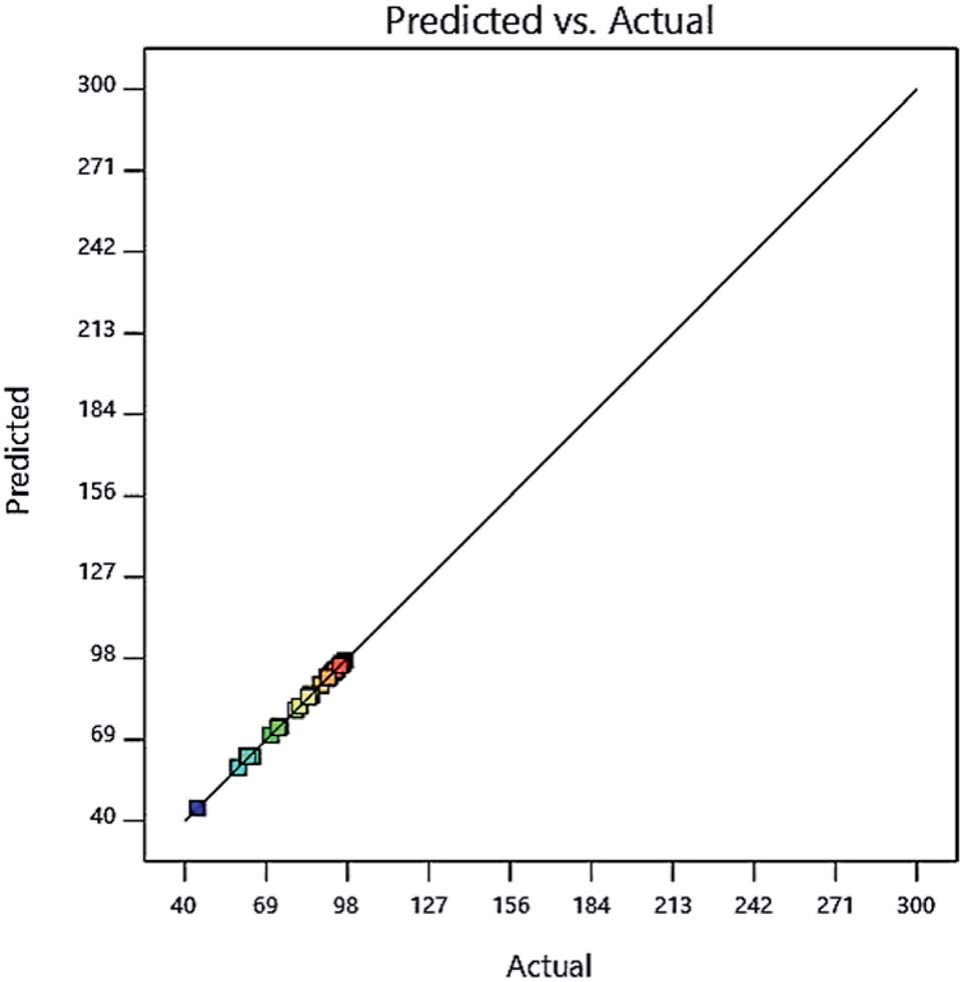
The amount of Cd(ii) adsorption is synthesized in the prediction model on the adsorbent.

In the ANOVA results, the higher the *F* value and lower the *P* value tend to, the model data will be more accurate and reliable. By examining Table 4, it is shown that the *F* value is high and very acceptable and the value of the parameter *P* is also low and acceptable. To demonstrate the fitting of the predicted model with experimental data, it can be shown by plotting the experimental data *versus* the model data of their compliance rate, as shown in Fig. 6. As shown in Fig. 6, the proposed model has very high compliance with the experimental data, so that the model (*R*^2^) is more than 0.99. Fig. 6 shows that model data have high power to predict the behavior of independent variables in the experiment, which confirms the ANOVA results. This high correlation and high predictive power help us to determine the best model for choosing the four variables in the experiment and the optimal amount of each, so that we can have the best removal percentage. The optimal amount and the best choice for the four independent variables are shown in Table 5. Also, the high correlation between experimental data and experimental data indicates that the Central Composite Design (CCD) method is applicable and powerful tool for predicting and selecting the best condition for independent variables.

**Table 5:** Optimum values for 4 independent variables, (*a*) initial metal concentration, (*b*) pH, (*c*) absorption time, (*d*) absorbent amount

Independent variables	(*a*) Initial metal concentration (mg L^−1^)	(*b*) pH	(*c*) Absorption time (min)	(*d*) Absorbent amount (gr)
Optimum amount	502	5	63	0.18

The initial value of the metal ion concentration (*a*), pH (*b*), the adsorption time (*c*) and adsorbent amount (*d*) For adsorbent MnFe_2_O_4_-TiO_2_-UIO-66 was 502 (mg l^−1^), 5, 63 (min) and 0.18 (gr) respectively.

### 3.2 Effect of initial metal concentration

**Fig. 7:**
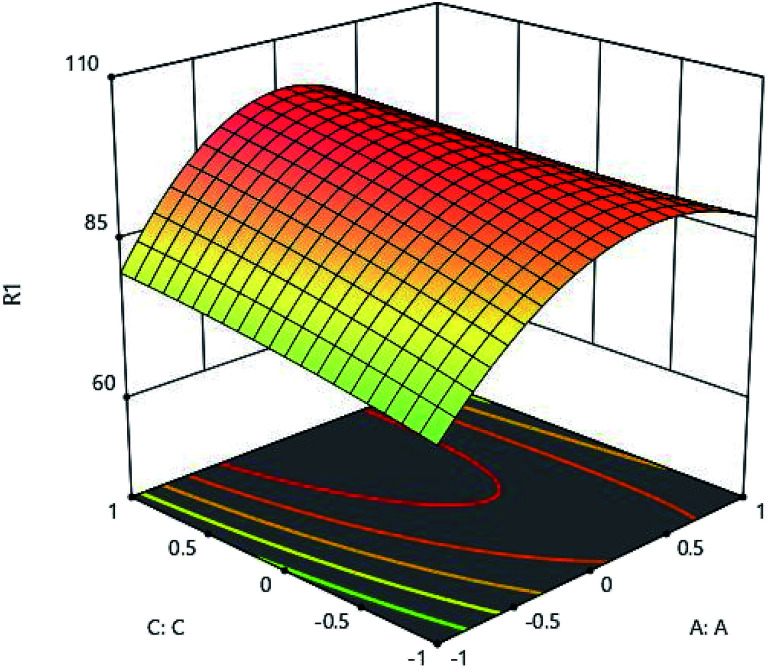
Effect of initial metal concentration (A:A) and absorption time (C:C) on the Cd(ii) adsorption by synthesized adsorbent.

**Fig. 8:**
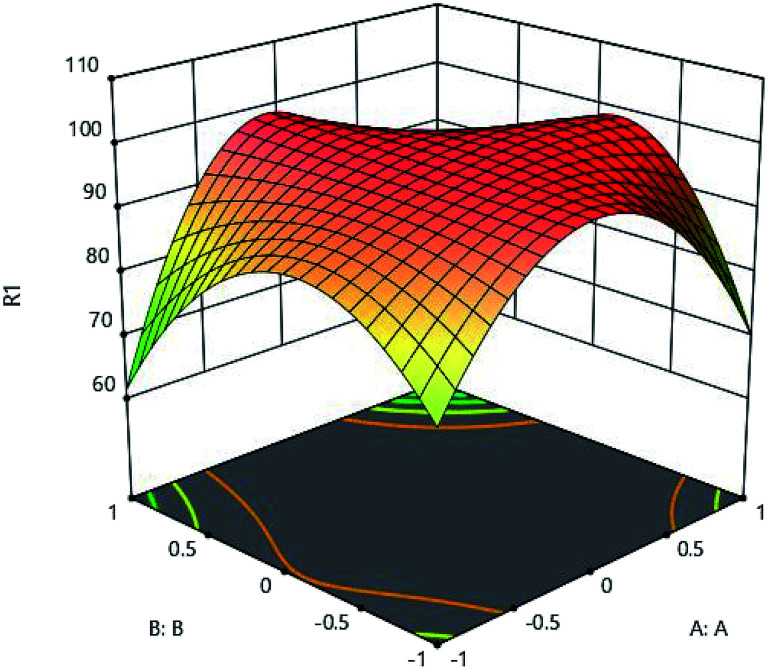
Effect of initial metal concentration (A:A) and pH (B:B) on the Cd(ii) adsorption by synthesized adsorbent.

The results of initial metal concentration and adsorption time are shown in Fig. 7. To evaluate the effects of initial metal concentration and metal adsorption capability on the adsorbent, the initial concentrations of metal were considered from 160 to 960 (mg l^−1^). The results of the initial metal concentration and adsorbent amount are shown in Fig. 8. As shown in Fig. 7 and 8, a significant amount of metal was absorbed in short time with a low adsorbance. This can be due to the ratio of the total activity of adsorbent sites to all metal ions in solution. High amount of metal ions is likely to adsorb because of its high adsorption level leading to the high interaction of metallic ions with adsorbent. On the other hand, the high concentration of metal ions in the solution causes the active sites to be absorbed on the adsorbent and the amount of adsorption decreases after a while, these phenomena can be observed clearly in these figures. Specifically, after increasing the concentration of metal ions, a certain degree is observed due to the competition and filling of the adsorbent surface and the active sites, as a result of which the adsorption decreases. This is very low change and very precise measurements can record it to a small extent;^16,17^ therefore, statistical analysis showed that, the interaction between the initial concentrations of the metal ions and the adsorbent has a significant effect, so that the initial concentration of the metal has a significant effect on the adsorption rate. From another aspect, the accumulation and compression of the adsorbent in the solution decreases the effective adsorption level as a result of the adsorption rate.^18^ The initial concentration of metal ions acts as an important driving force in overcoming the mass transfer resistance between the two solid and liquid phases. As a result, the adsorption rate decreases in lower concentrations of metal ions and, in the high concentrations due to the adsorption of a specific surface area and excessive accumulation of metal on the adsorbent resulting in a repulsive effect, it leads to a reduction in adsorption percentage. For this reason, here the optimal value was specified for both parameters.

### 3.3 Effect of pH

**Fig. 9:**
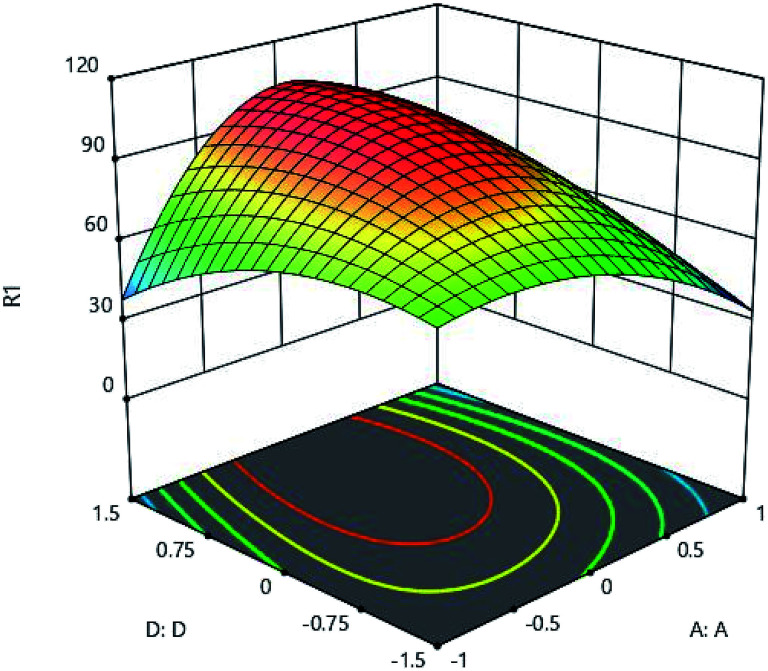
Effect of initial metal concentration (A:A) and adsorbent (D:D) on the Cd(ii) adsorption by synthesized adsorbent.

The effects of initial metal concentration and pH on the adsorption are shown in Fig. 9. pH of the aqueous solution is one of the important parameters greatly influencing the adsorption process, which has a significant effect on the adsorption surface charge and degree of ionization. Fig. 9 clearly shows the effect of adsorbents' pH. To determine the effect of pH on adsorption, environmental pH of the experiment was changed from 1 to 9. The results of analysis showed that, in low levels of pH, the adsorption rate was very low and until the optimal pH, the rate of adsorption increases dramatically, and then it decreases. According to the results of analysis, the optimum pH was obtained as 5. By changing the pH of the solution, the surface profile of the adsorbent is modified and improves the adsorption process.^19^ The adsorption process is also preferred to take place at acidic pH, because acidity of the soluble environment has a positive effect on the binding of metal ions to active sites on the adsorbent.^18^ When the pH of the solution rises, and along with movement of the solution medium toward the alkalization, OH-ion is created, causing competition between these ions and metal ions,significantly reducing the amount of adsorption.^20^

### 3.4 Effect of adsorption time

**Fig. 10:**
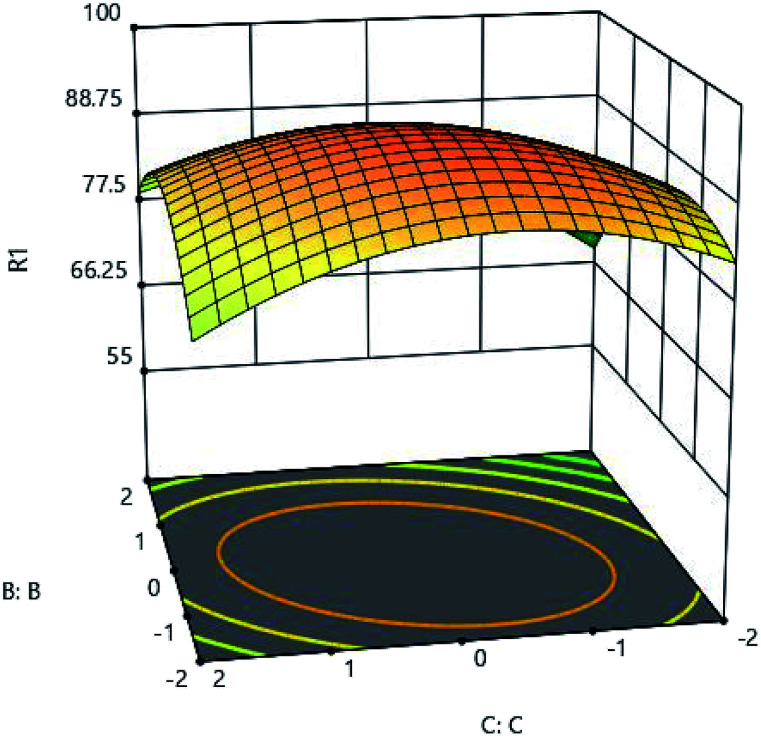
The effect of adsorption time (C:C) and pH (B:B) on the Cd(ii) adsorption by synthesized adsorbent.

To determine the effects of contact time, the range between 24 and 120 minutes was considered for testing. Fig. 7 and 10 show the effect of time. As shown in these figures, it is well established that the adsorption rate is significant in the first contact of the adsorbent and the metal, and the high adsorption rate at the primary time is due to the high amount of adsorbent on the active sites, then this adsorption capacity decreases and progresses well until it eventually remains almost constant. Adsorption characteristics such as adsorbent structure depends on the nature of the equilibrium.

According to the results, the synthesized adsorbent structure was found to be very suitable for adsorption of cadmium metal ions. As mentioned, the adsorption process can be divided into two different steps in terms of adsorption rate. In the first step, there is a high rate of adsorption in a short time, but in second step, adsorption rate is low leading to the long time for adsorption. The high rate of adsorption in the first stage is due to the large number of active sites on the adsorbent which all of them are free of charge to be filled rapidly by metal ions, and the slowdown in adsorption can be due to the problems that metal ions encounter when absorbed by the adsorbent. Because at this stage, metal ions are trying to fill the remaining active sites on the adsorbent, which will take longer, as well as the repulsion force created by the metal ions in this case.^21,22^ The results of the analysis revealed that, the most optimal adsorption time was equal to 63 minutes.

As indicated later, the rate of adsorption greatly reduces and will not increase dramatically. The results of analysis and comparison of the synthesized adsorbent with other adsorbents expressed in certain sources showed that, the adsorbent velocity is very high for adsorbing the contaminated metal and can adsorb high amounts at low rates, and eventually it is very low until the adsorption can be continued.

### 3.5 Effect of adsorbent amount

**Fig. 11:**
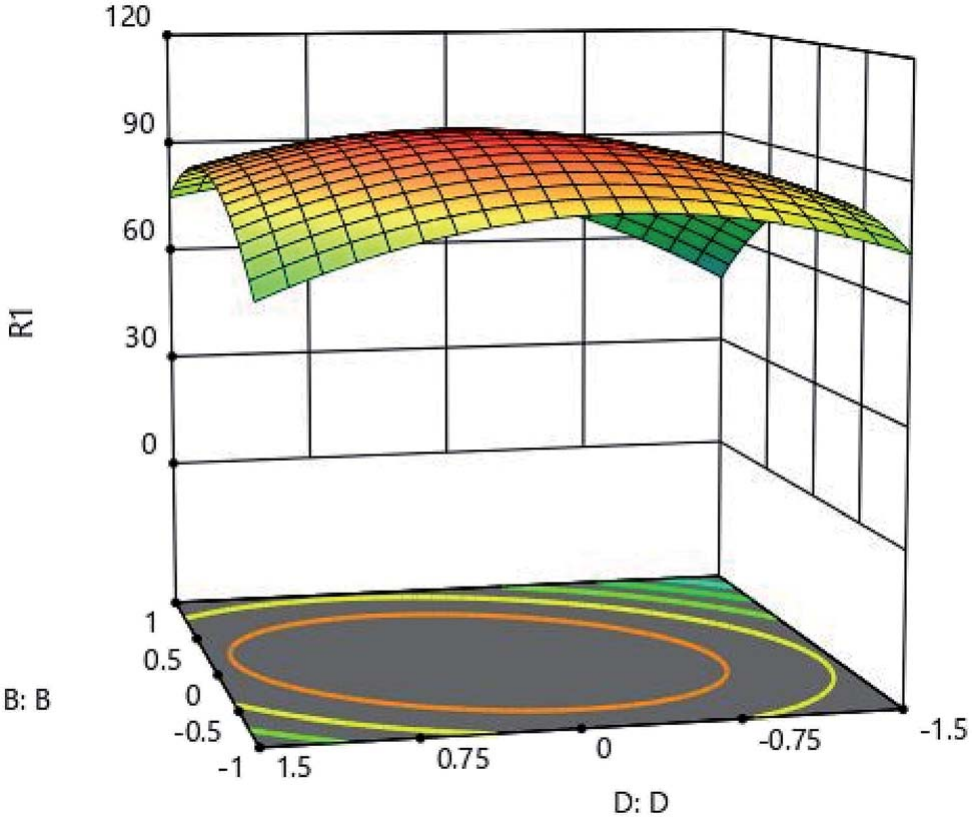
Effect of adsorbent amount (D:D) and pH (B:B) on the Cd(ii) adsorption by synthesized adsorbent.

To investigate the effect of adsorbent ion, adsorbent amount was studied in the range of 0.05 to 0.25 grams. As shown in Fig. 9 and 11, very low adsorption in the solution results in a high adsorption of the metal, and the adsorbent is well adsorbed by the material itself. The maximum adsorption capacity of 502 (mg l^−1^) was obtained for 0.18 grams of adsorbent, and the increase in adsorbent content did not have a very beneficial effect, and practically did not increase the adsorption rate significantly with more adsorbent adsorption.

This behavior can occur due to the massive adsorption of the solution as a result of which the amount of adsorbent increases in the solution. This phenomenon will reduce the active sites on the adsorbent and influence the adsorption capacity. In fact, increasing the amount of adsorbent only leads to more adsorbent consumption and does not have much effect on adsorption.^22–26^ The adsorbent mass in the solution also reduces the level of the adsorbent.^28^ This phenomenon occurring as a result of an increase in adsorption concentration, also reduces penetration along its path, leading to a slight decrease in adsorption.^27,28^

### 3.6 Isotherm of adsorption

For analysis of adsorption data, Langmuir isotherms (eqn (6)) and Freundlich (eqn (7)) were used.6
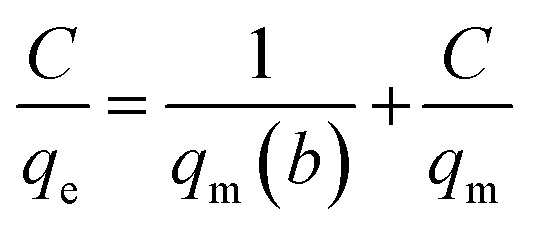
7
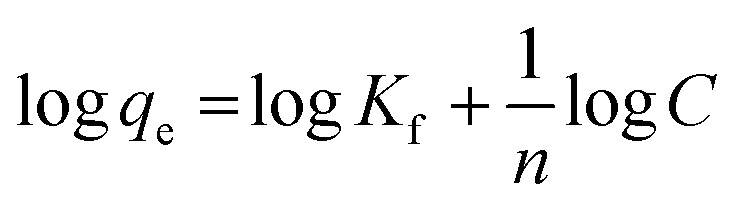
where, *q*_e_ is the adsorption rate of Cd(ii), *C* is the equilibrium concentration in adsorption (mg g^−1^), and *b* is Langmuir constant (l mg^−1^). *q*_m_ is the maximum amount of monolayer adsorption capacity, *K*_f_ (mg g^−1^) and *n* (L mg^−1^) are the Freundlich constants. The experimental data of the adsorption at constant temperature and concentration were plotted in eqn (5) and (6) and the equations were calculated by the slope of the fitted line. Finally, the data obtained from these equations are used to understand how adsorbent metal is adsorbed on the adsorbent. By drawing this curve on the amount basis of the metal adsorbed on adsorbent *versus* the amount of metal dissolved in a solution at constant temperature, the equilibrium behavior of the adsorption process can be investigated. For a better examination of Langmuir isotherm, a dimensionless number (*R*_L_) can be used. If it is between 0 and 1, then the choice of Langmuir isotherm is suitable. To calculate (*R*_L_), we can use the constant b and the initial concentration of the metal using eqn (7). By analyzing the correlation of the results obtained from the Freundlich and Langmuir isotherm, it is possible to decide whether the adsorbent adsorption is single-layered or multilayer. However the correlation obtained for *R*^2^ was higher for Langmuir isotherm, it can be concluded that adsorption was monolayer and homogeneous. However, this correlation and *R*^2^ were higher, it can be concluded that the adsorption has been multi-layered and non-homogeneous. The results obtained for the data (*R*_L_) in Table 6 and the Langmuir and Freundlich isotherm results are presented in Table 7.8
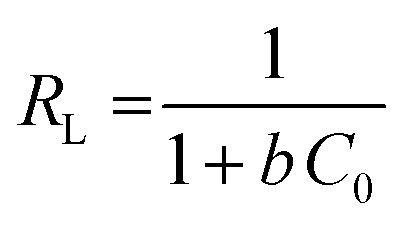


**Table 6:** *R*_L_ factor for synthesized adsorbent

Adsorbent	*R*_L_
UIO-66-MnFe_2_O_4_-TiO_2_	0.89
	0.91
	0.912
	0.94
	0.96

**Table 7:** Langmuir and Freundlich isotherm variables for synthesized adsorbents

UIO-66-MnFe_2_O_4_-TiO_2_	
**Langmuir isotherm**	
*q*_max_	1036.4
*b*	0.0381
*R* ^2^	0.996

**Freundlich isotherm**	
1/*n*	1.628
*K*_f_	4.568
*R* ^2^	0.913

By examining *R*^2^ data for Langmuir and Freundlich isotherm, Langmuir isotherm fit for adsorbent with *R*^2^ is 0.996 for optimum and shows better correlation. So, it is determined that the adsorption of metal on the adsorbent is monolayer and homogeneous. The witness can also see that the *R*_L_ is less than 1, and that the value of 1/*n* is greater than 1 in the Freundlich isotherm. As a result, Langmuir's isotherm equation has a better adherence to it. As summarized in Table 8, the maximum adsorption capacity (*Q*_max_) of UIO-66-MnFe_2_O_4_-TiO_2_ toward Cd(ii) which calculated from the Langmuir isotherm model was compared with those of other adsorbents from different sources.^29–35^

**Table 8:** Comparison for the adsorption of Cd(ii) by different adsorbents from aqueous solutions

Absorbent	Heavy metal	pH	Contact time (min)	*Q*_max_ (mg g^−1^)	Reference
Polyurethane ethylene-shoe material	Cd(ii)	5	881	396	27
Fe_3_O_4_-En-MIL-101	Cd(ii)	5	—	198	28
NH_2_-Zr-MOFs	Cd(ii)	—	120	177	29
FJI-H12	Cd(ii)		50	439	30
UiO-66-NH_2_ MOF into the PAN/chitosan nanofibers	Cd(ii)	—	—	355	31
Fe_3_O_4_@TAR	Cd(ii)	—	—	210	32
Aminated PAN/chitosan/rectorite fibers	Cd(ii)	6	30	714	33
UIO-66-MnFe_2_O_4_-TiO_2_	Cd(ii)	5	63	783	This study

### 3.7 Investigation of adsorption kinetics

In order to find a detailed view of the adsorption reaction mechanism, we need to have a thorough study of the adsorption kinetics in different concentrations. To study the adsorption kinetics, two first-order and quasi-quadratic kinetic models were used. The quasi-first-order Lagrange model kinetics is applicable to the adsorption of the soluble material physically, indicating a poor interaction between metal ions and adsorbents. While the pseudo-second order model is based on assumption of adsorption with chemical reaction. The equations of the first-order and pseudo-second-order models are presented in eqn (9) and (10), respectively.9
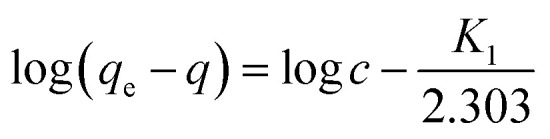
10
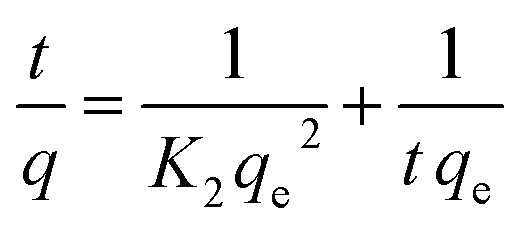
Here *q*_e_ is the adsorption capacity (mg g^−1^) at the time of equilibrium and *q* the adsorption capacity (mg g^−1^) at time *t* (min). *K*_1_ is the constant coefficient of the pseudo-first-order model (l min^−1^). *K*_2_ is the constant coefficient of the pseudo-second order model (g mg^−1^ min^−1^). Adsorption kinetics studies were carried out using two models. The correlation between laboratory data and model with *R*^2^ is shown in Table 9. By analyzing the data in Table 8, a pseudo-second order model with higher correlation and *R*^2^ of more than 0.99 is preferable to describe the kinetics of adsorption. With regard to the higher correlation generated in the pseudo-second order model, it can be predicted that the adsorption of metal on the adsorbent is carried out chemically.

**Table 9:** Variables of pseudo-first order & pseudo-Second order-model equations

UIO-66-MnFe_2_O_4_-TiO_2_	
**Pseudo-first order-model**	
*q*_e_	572
*K*_1_	0.326
*R* ^2^	0.902

**Pseudo-second order-model**	
*q*_e_	694
*K*_2_	0.394
*R* ^2^	0.996

By examining the parameters *R*^2^ and *R*_L_ and 1/*n*, it can be well established that the best model for predicting and fitting the adsorption kinetics of the pseudo-second order model. By this expression it is determined that adsorption is carried out chemically and heterogeneously.

### 3.8 Investigation of adsorbent before and after adsorption of Cd(ii)

**Fig. 12:**
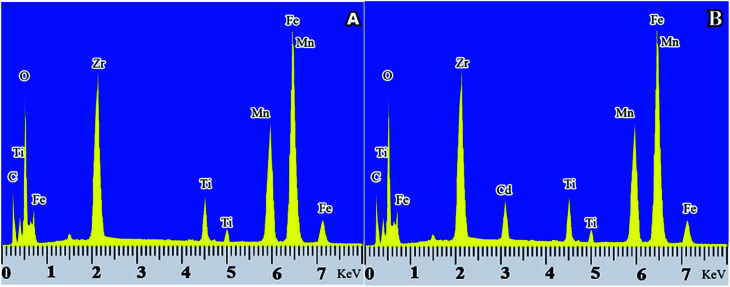
EDX spectrum of the adsorbent sample synthesized before and after absorption of Cd(ii), (A) of the EDX spectrum before adsorption of Cd(ii), (B) of the EDX spectrum after absorption of Cd(ii).

To investigate the adsorption of Cd(ii) onto the adsorbent, a sample of adsorbent was synthesized before and after adsorption of Cd(ii) for EDX study. Fig. 12(a) shows the EDX spectrum prior to adsorption, and Fig. 12(b) shows the EDX spectrum after adsorption of Cd(ii). As it is well-defined in the figure, prior to capture, Cd(ii) courier is not seen but after adsorption, the peak of Cd(ii) appears in its place. The spectroscopy, in addition to specifying the synthesis of adsorbent and confirming its high purity indicated the successful adsorption of contaminants on the adsorbent surface.

**Fig. 13:**
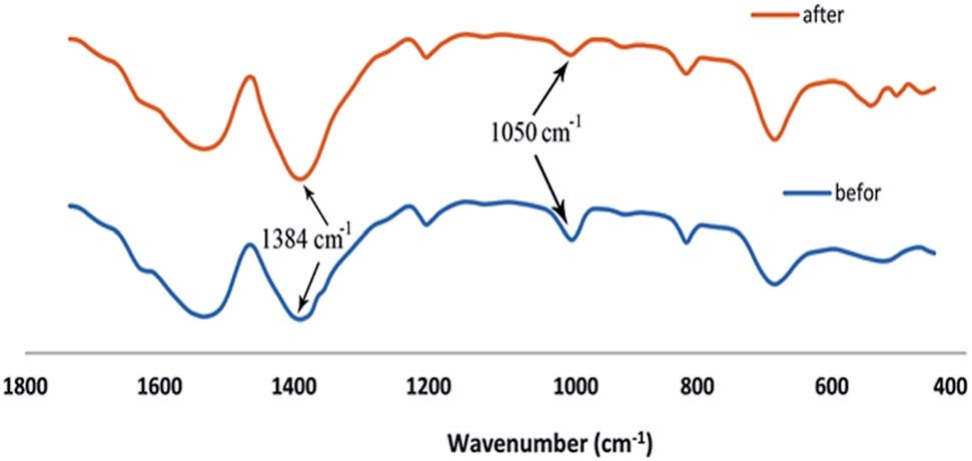
The UIO-66-MnFe_2_O_4_-TiO_2_ FT-IR after and before Cd(ii) adsorption.

The FT-IR technique was used to better understand the process of adsorption Cd(ii) onto the synthesized adsorbents as shown in Fig. 13. For this reason, the FT-IR spectra were taken from UIO-66 before and after Cd(ii) adsorption. Comparison of FTIR results before and after adsorption of Cd(ii) showed new significant peaks and the loss of a number of peaks. The reduction of peak strength at 1055 cm^−1^ is due to a decrease in the number of Zr–O–H bonds. It can be concluded that, the formation of Zr–O–Cd bonds reduced Zr–O–H bonds. On the other hand, the increase of peak strength at 1384 cm^−1^ can be attributed to the NO_3_ produced from the source of cadmium nitrate due to the release of Cd^2+^ ion in the environment. Increase in the peaks strength and new peaks created in the range of 400–600 cm^−1^ is related to the Cd–O peaks. Results of FT-IR showed that, the adsorption of Cd(ii) is done chemically, and it can be assumed that Cd(ii) is located instead of O–H bonds in UIO-66.

### 3.9 Investigating desorption capability and reusing the adsorbent in adsorption of Cd(ii)

**Fig. 14:**
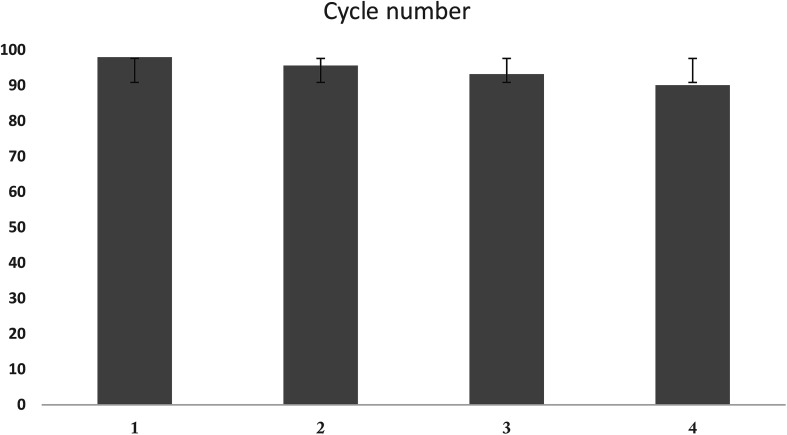
Number of absorbent recovery cycles.

The ability to reuse the adsorbent is one of the key and economic parameters regarding the use of adsorbent. Therefore, the ability of UIO-66-MnFe_2_O_4_-TiO_2_ recovery during the Cd(ii) adsorption process was investigated. For recovering the adsorbent, 1 gr of adsorbent was poured to 250 ml of 0.01 molar sodium nitrate solution after adsorption of Cd(ii) ions, and was stirred for 24 hours using a magnetic stirrer, and this work was repeated for three times. Finally, to determine the adsorption capacity of the recovered adsorbent in optimal conditions, Cd(ii) ions were adsorbed. The results are presented in Fig. 14.

## 4 Conclusion

In the present study, the adsorption of Cd(ii) in a magnetic nanoparticle composite (UIO-66-MnFe_2_O_4_-TiO_2_) using a metal–organic framework was investigated with good surface efficiency of 792 m^2^ g^−1^ and magnetic property of 22 emu g^−1^ of the synthesized gases. Then, RSM and CCD method were optimized. For initial metal concentration and optimal adsorbent content, the optimum adsorption conditions were obtained as 502 mg l^−1^ and 0.18 grams, respectively. The optimum pH and time were obtained at 5 and 63 minutes, respectively. The study of kinetics and isotherm adsorption showed that, the second-order kinetic model and Langmuir isotherm are well covered by Cd(ii) adsorption data, implying that adsorption has been synthesized homogeneously and chemically on the adsorbent.

At optimum conditions, the maximum adsorption rate of Cd(ii) was obtained as 98%. Comparing the synthesized adsorbent with those mentioned in the previous studies showed that, adsorption of other heavy metals is possible using the synthesized adsorbents. Also, the adsorbent separation conditions were investigated after metal adsorption. The results showed that, adsorbent is well able to isolate from the solution after adsorption.

## Conflicts of interest

There are no conflicts to declare.

## Supplementary Material

## References

[cit1] Babarinde A., Ogundipe K., Sangosanya K. T., Akintola B. D., Elizabeth Hassan A.-O. (2016). Comparative study on the biosorption of Pb (II), Cd (II) and Zn (II) using Lemon grass (Cymbopogon citratus): kinetics, isotherms and thermodynamics. Chem. Int..

[cit2] Bian B., Lv L., Yang D., Zhou L. (2014). Migration of heavy metals in vegetable farmlands amended with biogas slurry in the Taihu Basin, China. Ecol. Eng..

[cit3] Ammari T. G., Al-Labadi I., Tahboub A., Ghrair A. (2015). Assessment of unmodified wetland bio-waste: Shoots of Cyperus laevigatus, for cadmium adsorption from aqueous solutions. Process Saf. Environ. Prot..

[cit4] Rady M. M., Hemida K. A. (2015). Modulation of cadmium toxicity and enhancing cadmium-tolerance in wheat seedlings by exogenous application of polyamines. Ecotoxicol. Environ. Saf..

[cit5] Singh P. K., Kumar Shrivastava A., Chatterjee A., Pandey S., Rai S., Singh S., Rai L. C. (2015). Cadmium toxicity in diazotrophic Anabaena spp. adjudged by hasty up-accumulation of transporter and signaling and severe down-accumulation of nitrogen metabolism proteins. J. Proteomics.

[cit6] Meitei M. D., Prasad M. N. V. (2014). Adsorption of Cu (II), Mn (II) and Zn (II) by Spirodela polyrhiza (L.) Schleiden: equilibrium, kinetic and thermodynamic studies. Ecol. Eng..

[cit7] Meitei M. D., Prasad M. N. V. (2014). Adsorption of Cu (II), Mn (II) and Zn (II) by Spirodela polyrhiza (L.) Schleiden: equilibrium, kinetic and thermodynamic studies. Ecol. Eng..

[cit8] Hubadillah S. K., Othman M. H. D., Ismail A. F., Rahman M. A., Jaafar J., Iwamoto Y., Honda S., Dzahir M. I. H. M., Yusop M. Z. M. (2018). Fabrication of low cost, green silica based ceramic hollow fibre membrane prepared from waste rice husk for water filtration application. Ceram. Int..

[cit9] Kitagawa S., Matsuda R. (2007). Chemistry of coordination space of porous coordination polymers. Coord. Chem. Rev..

[cit10] Ahmed I., Sung H. J. (2014). Composites of metal–organic frameworks: preparation and application in adsorption. Mater. Today.

[cit11] Jiang X., Chen H.-Y., Liu L.-L., Qiu L.-G., Jiang X. (2015). Fe_3_O_4_ embedded ZIF-8 nanocrystals with ultra-high adsorption capacity towards hydroquinone. J. Alloys Compd..

[cit12] Zhong X., Yang J., Chen Y., Qiu X., Zhang Y. (2015). Synthesis of magnetically separable MnFe_2_O_4_ nanocrystals *via* salt-assisted solution combustion method and their utilization as dye adsorbent. J. Ceram. Soc. Jpn..

[cit13] Nasehi P., Saei Moghaddam M., Abbaspour S. F., Karachi N. (2019). Preparation and characterization of a novel Mn-Fe_2_O_4_ nanoparticle loaded on activated carbon adsorbent for kinetic, thermodynamic and isotherm surveys of aluminum ion adsorption. Sep. Sci. Technol..

[cit14] Ghosh P., Colón Y. J., Snurr R. Q. (2014). Water adsorption in UiO-66: the importance of defects. Chem. Commun..

[cit15] Xiao H.-M., Liu X.-M., Fu S.-Y. (2006). Synthesis, magnetic and microwave absorbing properties of core-shell structured MnFe_2_O_4_/TiO_2_ nanocomposites. Compos. Sci. Technol..

[cit16] AjayKumar A. V., Darwish N. A., Hilal N. (2009). Study of various parameters in the biosorption of heavy metals on activated sludge. World Appl. Sci. J..

[cit17] Yari S. (2015). *et al.*, Adsorption of Pb (II) and Cu (II) ions from aqueous solution by an electrospun CeO_2_ nanofiber adsorbent functionalized with mercapto groups. Process Saf. Environ. Prot..

[cit18] AjayKumar A. V., Darwish N. A., Hilal N. (2009). Study of various parameters in the biosorption of heavy metals on activated sludge. World Appl. Sci. J..

[cit19] Garg U., Kaur M. P., Jawa G. K., Sud D., Garg V. K. (2008). Removal of cadmium (II) from aqueous solutions by adsorption on agricultural waste biomass. J. Hazard. Mater..

[cit20] Aksu Z., Alper Isoglu I. (2006). Use of agricultural waste sugar beet pulp for the removal of Gemazol turquoise blue-G reactive dye from aqueous solution. J. Hazard. Mater..

[cit21] Babarinde A., Ogundipe K., Sangosanya K. T., Akintola B. D., Elizabeth Hassan A.-O. (2016). Comparative study on the biosorption of Pb (II), Cd (II) and Zn (II) using Lemon grass (Cymbopogon citratus): kinetics, isotherms and thermodynamics. Chem. Int..

[cit22] Babarinde A., Onyiaocha G. O. (2016). Equilibrium sorption of divalent metal ions onto groundnut (Arachis hypogaea) shell: kinetics, isotherm and thermodynamics. Chem. Int..

[cit23] Iqbal M. J., Cecil F., Ahmad K., Iqbal M., Mushtaq M., Naeem M. A., Bokhari T. H. (2013). Kinetic Study of Cr (III) and Cr (VI) Biosorption Using Rosa damascena Phytomass: A Rose Waste Biomass. Asian J. Chem..

[cit24] Manzoor Q., Nadeem R., Iqbal M., Saeed R., Ansari T. M. (2013). Organic acids pretreatment effect on Rosa bourbonia phyto-biomass for removal of Pb (II) and Cu (II) from aqueous media. Bioresour. Technol..

[cit25] Ullah I., Nadeem R., Iqbal M., Manzoor Q. (2013). Biosorption of chromium onto native and immobilized sugarcane bagasse waste biomass. Ecol. Eng..

[cit26] Gong R., Ding Y., Liu H., Chen Q., Liu Z. (2005). Lead biosorption and desorption by intact and pretreated Spirulina maxima biomass. Chemosphere.

[cit27] Ofomaja A. E., Ho. Y.-S. (2007). Equilibrium sorption of anionic dye from aqueous solution by palm kernel fibre as sorbent. Dyes Pigm..

[cit28] Shukla A., Zhang Y.-H., Dubey P., Margrave J. L., Shukla S. S. (2002). The role of sawdust in the removal of unwanted materials from water. J. Hazard. Mater..

[cit29] Iqbal M., Iqbal N., Ahmad Bhatti I., Ahmad N., Zahid M. (2016). Response surface methodology application in optimization of cadmium adsorption by shoe waste: a good option of waste mitigation by waste. Ecol. Eng..

[cit30] Babazadeh M., Hosseinzadeh-Khanmiri R., Abolhasani J., Ghorbani-Kalhor E., Hassanpour A. (2015). Solid phase extraction of heavy metal ions from agricultural samples with the aid of a novel functionalized magnetic metal–organic framework. RSC Adv..

[cit31] Wang K., Gu J., Yin N. (2017). Efficient removal of Pb (II) and Cd (II) using NH_2_-functionalized Zr-MOFs *via* rapid microwave-promoted synthesis. Ind. Eng. Chem. Res..

[cit32] Liang L., Chen Q., Jiang F., Yuan D., Qian J., Lv G., Xue H., Liu L., Jiang H.-L., Hong M. (2016). In situ large-scale construction of sulfur-functionalized metal–organic framework and its efficient removal of Hg (II) from water. J. Mater. Chem. A.

[cit33] Jamshidifard S., Koushkbaghi S., Hosseini S., Rezaei S., Karamipour A., Irani M. (2019). Incorporation of UiO-66-NH_2_ MOF into the PAN/chitosan nanofibers for adsorption and membrane filtration of Pb (II), Cd (II) and Cr (VI) ions from aqueous solutions. J. Hazard. Mater..

[cit34] Ghorbani-Kalhor E. (2016). A metal-organic framework nanocomposite made from functionalized magnetite nanoparticles and HKUST-1 (MOF-199) for preconcentration of Cd (II), Pb (II), and Ni (II). Microchim. Acta.

[cit35] Huang M., Hu T., Chen J., Liu R., Liang Z., Jiang L., Shi X., Du Y., Deng H. (2018). Chitosan-rectorite nanospheres embedded aminated polyacrylonitrile nanofibers *via* shoulder-to-shoulder electrospinning and electrospraying for enhanced heavy metal removal. Appl. Surf. Sci..

